# A Rapid In Vivo Toxicity Assessment Method for Antimicrobial Peptides

**DOI:** 10.3390/toxics12060387

**Published:** 2024-05-25

**Authors:** Yulang Chi, Yunhui Peng, Shikun Zhang, Sijia Tang, Wenzhou Zhang, Congjie Dai, Shouping Ji

**Affiliations:** 1 College of Oceanology and Food Science, Quanzhou Normal University, Quanzhou 362000, China; ylchi@qztc.edu.cn (Y.C.);; 2 School of Advanced Manufacturing, Fuzhou University, Quanzhou 362200, China; 3 Academy of Military Medical Sciences, Beijing 100850, China; zhangskun@126.com; 4 School of Pharmacy, Quanzhou Medical College, Quanzhou 362011, China

**Keywords:** antimicrobial peptides, toxicity assessment, mouse acute lung injury model

## Abstract

Antimicrobial peptides (AMPs) represent a promising antibiotic alternative to overcome drug-resistant bacteria by inserting into the membrane of bacteria, resulting in cell lysis. However, therapeutic applications of AMPs have been hindered by their ability to lyse eukaryotic cells. GF-17 is a truncated peptide of LL-37, which has perfect amphipathicity and a higher hydrophobicity, resulting in higher haemolytic activity. However, there is no significant difference in the cytotoxicity against human lung epithelial cells between the GF-17 and LL-37 groups, indicating that there are significant differences in the sensitivity of different human cells to GF-17. In this study, LL-37 and GF-17 were administered to mouse lungs via intranasal inoculation. Blood routine examination results showed that LL-37 did not affect the red blood cells, platelet, white blood cells and neutrophil counts, but GF-17 decreased the white blood cells and neutrophil counts with the increasing concentration of peptides. GF-17-treated mice suffer a body weight loss of about 2.3 g on average in 24 h, indicating that GF-17 is highly toxic to mice. The total cell counts in the bronchoalveolar lavage fluid from GF-17-treated mice were 4.66-fold that in the untreated group, suggesting that GF-17 treatment leads to inflammation in the lungs of mice. Similarly, the histological results showed the infiltration of neutrophils in the lungs of GF-17-treated mice. The results suggest that the administration of GF-17 in the lungs of mice does not affect the red blood cells and platelet counts in the blood but promotes neutrophil infiltration in the lungs, leading to an inflammatory response. Therefore, we established a mouse acute lung injury model to preliminarily evaluate the in vivo toxicity of AMPs. For AMPs with a clinical application value, systematic research is still needed to evaluate their acute and long-term toxicity.

## 1. Introduction

The marked growth and global expansion of antibiotic resistance has been a severe clinical problem, which seriously endangers public health due to the eroded therapeutic benefit of conventional antibiotics and the lack of new antibiotics, such that we risk entering a post-antibiotic era. According to a recent report, approximately 700,000 people die as a direct result of multidrug-resistant (MDR) pathogen infections worldwide every year, and they carry an important economic burden [1,2]. It has been predicted that the global death toll caused by MDR pathogens will be 10 million people every year, with a loss of up to USD 100 trillion to the global economy by 2050 [3], as stated by the World Health Organization (WHO) in 2019 [4]. In fact, the WHO has named antibiotic resistance one of the three most important public health threats of the 21st century [5]. The major antibiotic-resistant bacteria are known as “ESKAPE”, which includes six highly virulent and antibiotic-resistant bacterial pathogens: *Enterococcus faecium*, *Staphylococcus aureus*, *Klebsiella pneumoniae*, *Acinetobacter baumannii*, *Pseudomonas aeruginosa*, and *Enterobacter* spp., which make the front-line if antibiotics fail [6,7,8]. Generally, it is difficult to obliterate antibiotic-resistant bacteria mainly due to gene mutation, acquiring exogenous genetic material, a decrease in membrane permeability, overexpression of the efflux pump, change in the target site, the presence of inactive subpopulations, biofilm formation, and enzymatic degradation [9,10]. Therefore, it is imperative to find new antibacterial drugs with a unique antibacterial mechanism to combat multidrug-resistant (MDR) bacterial infections.

Antimicrobial peptides (AMPs) are host defence molecules of the innate immunity of all living organisms. They are highly diverse in their amino acid sequences with a net-positive charge that attracts them to the generally negatively charged membranes of bacteria through electrostatic interaction. Upon contact with the cell membranes, they can fold into amphipathic conformations. Then, the hydrophobic face of the peptides facilitates insertion into the membrane to increase its permeability and disrupt its integrity, resulting directly or indirectly in cell lysis [11,12]. In addition, AMPs may also have more complex mechanisms, including interactions with an array of intracellular target molecules to induce metabolic and translational inhibition [13,14]. The stereotypical mechanism and pharmacodynamics of AMPs make it theoretically and clinically difficult for bacteria to evolve resistance. However, therapeutic applications of these AMPs have been hindered by their toxicity or their ability to lyse eukaryotic cells.

Physicochemical parameters believed to be important for the activity of AMPs have been identified, including peptide length, net charge, hydrophobicity and a secondary structure. Novel technologies, such as isomerisation, peptide lipidation, glycosylation, cyclisation, and other forms of biomimetic terminal modification and multimerisation [15,16,17], were applied for AMP engineering to improve their stability, activity and targetability. To date, the AMP database [Data Repository of Antimicrobial Peptides (DRAMP), http://dramp.cpu-bioinfor.org (accessed on 29 April 2024) currently contains 22,649 entries, 6107 of which are general AMPs (containing natural and synthetic AMPs), 16,229 patent AMPs and 96 AMPs in drug development (preclinical or clinical stage) [18]. However, none of them were approved for antibacterial treatment by the Food and Drug Administration (FDA).

The eukaryotic cytotoxicity is the biggest obstacle for AMPs’ clinical application. The traditional methods for evaluating the eukaryotic cytotoxicity of AMPs are hemolysis assays or cell viability assays at the cellular level, such as MTT, CCK8, and lactate dehydrogenase (LDH) release assays. However, the in vitro toxicity test cannot fully represent its toxicity in vivo. Establishing a method for the rapid and comprehensive evaluation of antimicrobial activity and toxicity of AMPs in vivo is of great help for the development of candidate AMPs for clinical application. 

The in vitro toxicity evaluation methods of antimicrobial peptides include the hemolysis test, cell activity test (MTT/CCK8/lactate dehydrogenase (LDH) release) and other methods. The in vivo evaluation method is mainly the median lethal dose, and the median lethal dose is usually administered through intravenous or intraperitoneal injection. Since antimicrobial peptides are mostly applied locally, this does not reflect the local reaction of antimicrobial peptides, and intraperitoneal injection often leads to the local accumulation of drugs at the point of injection. The concentration is too high, and after directly acting on the skin, it is impossible to obtain suitable samples for other tests except for pathological testing; the processing of pathological samples is cumbersome; and the interpretation of results is highly subjective. In this paper, we describe a method that can quantify the toxicity of antimicrobial peptides in vivo. Using different doses of antimicrobial peptides, intranasally administered to mice for 24 h, the state of the mice was observed, blood was collected and tested as routine analysis, and plasma was collected for ELISA testing. Bronchoalveolar lavage fluid was also examined for protein quantification, cell count and ELISA detection to comprehensively evaluate the side effects of antimicrobial peptides in vivo or their topical application and determine the concentration of antimicrobial peptides used in the method of evaluation.

## 2. Materials and Methods

### 2.1. Materials and Reagents

The LL-37 and GF-17 peptides were synthesised (Table 1) using the standard Fmoc procedure purified by reverse-phase semipreparative high-performance liquid chromatography, as described. The peptides were dissolved in PBS for a 1000 μmol/L stock solution for further use. The purity of the synthetic peptide was greater than 95%. Blood samples from healthy donors were obtained from the Beijing Red Cross Blood Centre. Human normal lung epithelial cells (BEAS-2B) and CCK8 were obtained from Beyotime Biotechnology (Shanghai, China). RPMI-1640 and FBS were from Sigma (St. Louis, MO, USA) and ExCell Bio (Shanghai, China), respectively. Three Gram-negative bacteria, *Escherichia coli* (1.8732), *Pseudomonas aeruginosa* (1.2421), and *Klebsiella pneumonia* (1.1736), and two Gram-positive bacteria, *Staphylococcus aureus* (1.8721), and *Staphylococcus epidermidis* (1.4260), were purchased from the China General Microbiological Culture Collection Centre (CGMCC, Beijing, China). All animals used in this experiment were bought from SPF (Beijing) Biotechnology Co., Ltd. (Beijing, China).

### 2.2. Antimicrobial Activity of the Peptides

The antibacterial activities of the peptides against three Gram-negative bacteria and three Gram-positive bacteria strains were measured using a modified version of the Clinical Laboratory and Standards Institute (CLSI) broth microdilution method, as described previously [19]. Briefly, bacteria were grown in a liquid LB medium (tryptone, 10 g/L, yeast extract, 5 g/L and NaCl, 10 g/L) at 37 °C to the mid-log phase, and the bacteria were diluted to 1 × 10^6^ colony-forming units (CFU)/mL. The peptides were serially diluted with phosphate-buffered saline (PBS), and 50 μL diluted peptides were added to 50 μL of the bacterial suspension in a 96-well plate (Corning Inc., Lowell, MA, USA). After incubation at 37 °C for 18 to 20 h, the absorbance of each well was recorded using a multi-well microplate reader (SpectraMax M5; Molecular Devices, Sunnyvale, CA, USA) at 600 nm. The lowest concentration at which the peptide inhibited the complete growth of the bacteria was taken as the minimal inhibitory concentration (MIC). 

### 2.3. Haemolytic Activity of the Peptides

The haemolytic activity of the peptides was assayed by a standard procedure with slight modifications [20]. Fresh human erythrocytes were washed three times and resuspended at 1.25% haematocrit in PBS. Forty microliters of the PBS-diluted peptide solution were added to a 96-well plate (Corning Inc., Lowell, MA, USA), and then 160 μL of erythrocytes were added. After incubation at 37 °C for 30 min, the samples were centrifuged, and the absorbance of the supernatant was measured at 450 nm using a multi-well microplate reader (SpectraMax M5; Molecular Devices, Sunnyvale, CA, USA) and compared with the 100% haemolysis caused by 0.1% Triton X-100. The percentage of haemolysis was calculated according to the equation.

### 2.4. Cytotoxicity of the Peptides

The toxicity of the peptides against BEAS-2B was assessed by a standard CCK-8 assay [21]. BEAS-2B was maintained in our laboratory. BEAS-2B cells were cultured in RPMI 1640 supplemented with 10% (*v*/*v*) FBS, 2 mM L-glutamine, 100 U/mL penicillin and 100 mg/mL streptomycin and maintained in a humidified incubator with 5% CO_2_ at 37 °C. BEAS-2B cells (5 × 10^3^ cells/well) were seeded in 96-well plates (Corning Inc., Lowell, MA, USA) and incubated overnight. Diluted peptides were added to BEAS-2B cells and incubated for 1 hour. Then, 10 μL of the CCK-8 solution was added and incubated for 1.5 hours. The absorbance was measured at 450 nm on a microplate reader (SpectraMax M5; Molecular Devices, Sunnyvale, CA, USA). Cell viabilities were calculated using the equation.

### 2.5. Mouse Acute Lung Injury (ALI) Model

All animals received care according to the guidelines outlined in the Guide for the Care and Use of Laboratory Animals. They were housed in an SPF-level breeding environment with a 12 h day and night cycle. The animal experiments were approved by the Experimental Animal Ethics Committee of the Academy of Military Medical Sciences. 

Acute lung injury (ALI) in vivo was established, as previously reported, with minor modification [22]. Briefly, mice were randomly divided into three groups (six mice for each group): the PBS treatment group, LL-37 group (administered with 40 μL LL-37 (2.4 mmol/L), P1 group (treated with 40 μL GF-17 (0.48 mmol/L) and P5 group (administered with 40 μL GF-17 (2.4 mmol/L). Mice were anesthetised with isoflurane, and 40 μL of the peptide was inoculated via the intranasal route. Mice were held upright for 1 min post-treatment and then placed into the cage for recovery. The body weight of the mice was measured using electronic scales before and 24 h after peptide treatment. The mice were sacrificed 24 h after the treatment. Bronchoalveolar lavage fluid (BALF) was collected as described below. The unilateral lung tissues were gently fixed in a buffered formaldehyde solution for histopathological assessment via hematoxylin and eosin (H&E) staining. Blood was collected for blood cell count.

### 2.6. Bronchoalveolar Lavage

BALF was collected as described previously [23]. In brief, the trachea and bronchi were exposed through a midline incision. The main bronchus was ligated, and the trachea was cannulated with a sterile 22-gauge Abbocath-T catheter (Abbott Laboratories, Sligo, Ireland). Unilateral, right-sided BAL was performed by instilling three 1 mL aliquots of sterile phosphate-buffered saline (PBS) and 0.7 to 0.9 mL of BAL fluid was retrieved per mouse. The number of total cells was enumerated by the automatic cell counter, then cells were spun down and the supernatant was used for protein concentration assay (Pierce BCA Protein Assay Kit).

### 2.7. Histology 

Hematoxylin-eosin (HE) staining sections of the paraffin-embedded tissues were used for histological analysis. After euthanising the mice, the intact lung was removed immediately. The samples were then fixed in 4% polyformaldehyde, paraffin-embedded and cut into 4 μm thick sections using a microtome (Leica Biosystems, Wetzlar, Germany). The sections were mounted on a glass slide and subjected to H&E staining. The representative mages were captured with Vectra 3.0.5 and processed with the Inform 2.2.0 (PerkinElmer, Waltham, MA, USA).

### 2.8. Statistical Analysis

Experimental data were encoded in Graphpad 6.0 (Boston, MA, USA) and presented as the mean ± SD. Statistical analyses were performed using unpaired *t*-tests and differences were considered significant at *p* < 0.05.

## 3. Results

### 3.1. Biophysical Properties of Peptides

**Figure 1:**
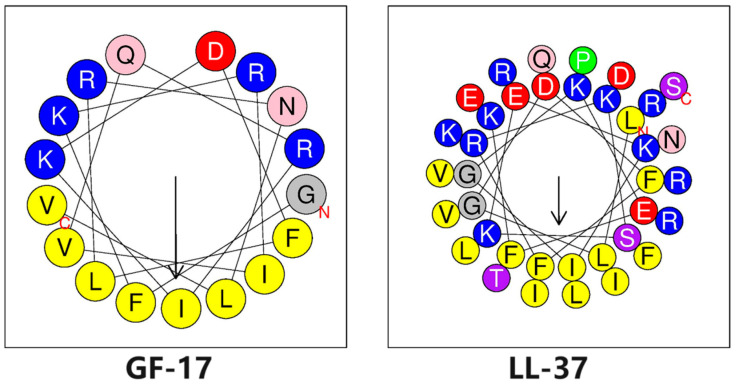
Helical wheel projections of GF-17 and LL-37. By default, the output presents the polar, positively charged amino acids as blue circles, the polar, negatively charged amino acids as red circles, the polar, uncharged amino acids as pink circles or purple circles, the hydrophobic residues as grey and green, and the most hydrophobic residues as yellow circles.

The LL-37 and GF-17 peptides were synthesised and purified by reverse-phase semipreparative high-performance liquid chromatography. The physicochemical properties of peptides were calculated by HeliQuest (https://heliquest.ipmc.cnrs.fr/cgi-bin/ComputParams.py, accessed on 28 April 2024). The helical wheel projections of LL-37 and GF-17 are shown in Figure 1, both of which can form an amphiphilic structure of a helix in which hydrophobic amino acid residues are concentrated on one side of the helix, while hydrophilic amino acid residues are concentrated on the other side. However, hydrophobic amino residues and hydrophilic amino acid residues are strictly arranged on both sides of the helix in GF-17, while the hydrophobic and hydrophilic surfaces of LL-37 are not as clearly defined as GF-17, and sometimes hydrophilic (hydrophobic) amino acid residues are arranged on the opposite side.

The sequences and biophysical characteristics of the peptides are listed in Table 1. The close agreement between the measured and theoretical molecular weights of LL-37 and GF-17 indicates that the compounds were synthesised to the desired specifications. Both LL-37 and its truncated peptide GF-17 [24] were entirely helical. However, compared to LL-37, GF-17 showed a higher value in its frequency of non-polar amino acid residues, hydrophobicity, mean relative hydrophobic moment (µH) and retention time measured by reverse-phase HPLC. Among them, the frequency of non-polar amino acid residues, hydrophobicity and retention time can reflect the relative hydrophobicity of α-helix and the hydrophobic distance of antimicrobial peptides is positively correlated with their amphiphilicity.

### 3.2. Antimicrobial Activity of Peptides

The antimicrobial activities of the peptides, indicated by the minimum inhibitory concentrations (MICs), were determined against a range of Gram-negative and Gram-positive bacterial strains (Table 2). The results show that LL-37 had a significant killing effect on Gram-negative bacteria, especially *Escherichia coli*, with an MIC of 1.56 μmol/L. However, the killing effect of LL-37 on Gram-positive bacteria was extremely limited, and the MIC was as high as 100 μmol/L. On the contrary, all strains were very sensitive to GP-17. The geometric mean of MIC values (GM) from the five strains was calculated to provide an overall evaluation of antimicrobial activity. The GM of GF-17 and LL-37 was 5.44 and 28.72, respectively. It was indicated that GF-17 had higher antimicrobial activity against a panel of bacterial species than LL-37. For Gram-positive bacteria (*S. aureus*, *S. epidermidis* and *K. pneumoniae*), the antibacterial effect increased by more than 16 times. The results indicate that GF-17 possesses strong bactericidal activity and cytotoxicity due to its high α-helical amphipathic structure [25]. In this study, GF-17 was chosen to evaluate the toxicity of antimicrobial peptides in vivo. 

### 3.3. Haemolytic Activity and Cytotoxicity of Peptides

**Figure 2:**
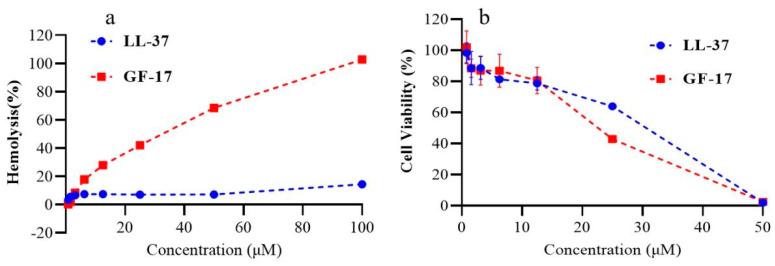
Haemolytic activity and cytotoxicity of LL-37 and GF-17. (**a**) Haemolytic activity of peptides. (**b**) Cell viability of BEAS-2B cells after LL-37 and GF-37 treatment.

The haemolytic activities of the peptides against human erythrocytes were determined as an indication of their toxicity towards mammalian cells. The results are summarised in Figure 2a. LL-37 exhibited lower haemolysis rates than GF-17(7.1% Vs 68.5% at concentrations of 50 μmol/L); even at concentrations of 100 μmol/L, the haemolysis rate of LL-37 was only 14.4%.

The therapeutic index (TI) is defined as the ratio of the minimum haemolytic concentration (MHC) of peptides to the GM of peptides, and this value is used to evaluate the cell selectivity of peptides towards the negatively charged region of bacterial cell membranes over zwitterionic mammalian cell membranes [26]. Similar to the results of the haemolytic activities of the peptides, GF-17 exhibited increased antimicrobial activity compared with LL-37. Taken together, LL-37 exhibited a higher therapeutic index compared with GF-17, which was 3.48 and 1.15, respectively. These results show that GF-17 increases both antibacterial activity and haemolytic activity, implying a narrow therapeutic window.

To further examine the toxicity of these peptides against mammalian cells, the CCK-8 assay was performed on BEAS-2B cells. This was similar to the results of the haemolytic assay. Unlike haemolysis analysis experiments, LL-37 exhibited similar toxicity to GF-17 (Figure 2b). For example, the viability of GF-17-treated and LL-37-treated cells was 56.5% and 64.0%, respectively, at concentrations of 25 μmol/L. When the peptide concentration was 50 μmol/L, the viability of GF-17-treated and LL-37-treated cells dramatically reduced to zero. The results indicate that different evaluation methods produce different results in vitro, and red blood cells are more sensitive to the cytotoxicity of antimicrobial peptides.

### 3.4. In Vivo Studies

**Figure 3:**
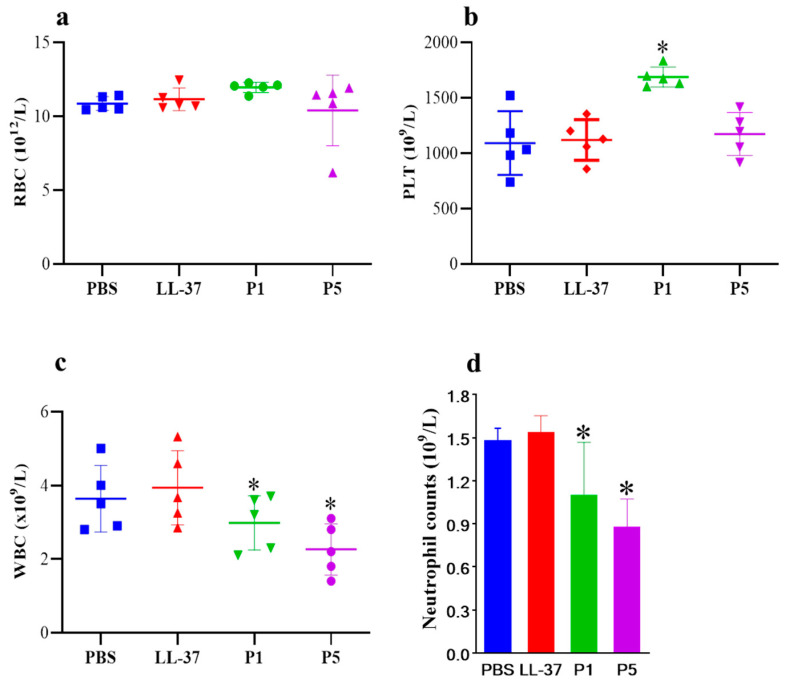
The blood routine of peptide-treated mice. (**a**) Red blood cell counts. (**b**) Platelet counts. (**c**) WBC counts. (**d**) Neutrophil counts. *, *p* < 0.05.

To validate the in vitro toxicity of antimicrobial peptides, we established a mouse ALI model via the intranasal inoculation of an antibacterial peptide into the lung. After inoculation, the blood routine of the mice was examined. As shown in Figure 3, there was no significant difference in red blood cell and platelet counts among the three experimental groups of mice (Figure 3a,b). However, in the GF-17 treatment groups, WBC counts decreased with increasing GF-17 dosage, and there was no significant difference in the WBC count between the LL-37 group and the PBS group. The WBC counts of PBS, LL-37, and groups P1 and P5 were 3.50, 3.67, 3.20 and 2.20 × 10^9^/L, respectively. Similarly, the neutrophil counts in the blood of GF-17-treated mice decreased with the increasing GF-17 concentration (Figure 3d), and there was no significant difference in the WBC count between the LL-37 group and the PBS group. The experimental results indicate that the intranasal infusion of GF-17 into the lungs of mice can reduce the number of immune-related cells in the blood of GF-17-treated mice.

**Figure 4:**
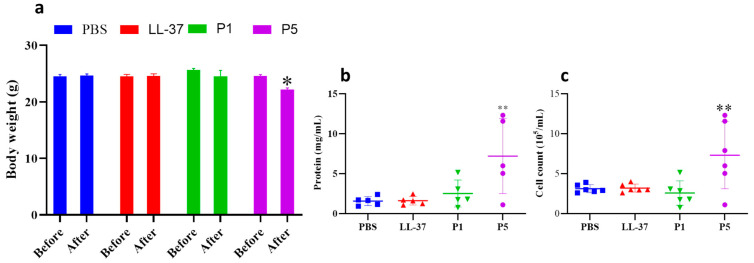
Body weight and the cells and protein in the BALF of GF-17-treated mice. (**a**) Body weight. (**b**) Protein concentration. (**c**) Cell counts. *, *p* < 0.05; **, *p* < 0.01.

Mice in group P5 (administered with 40 μL GF-17 (2.4 mmol/L) suffered a body weight loss of about 2.3 g on average (Figure 4), while no significant change in body weight was found in the LL-37 and P1 groups. The protein concentration of BALF in the P5 group was 3.32-fold of the untreated group, which was 2.2 and 7.3 mg/mL, respectively. Similar to the results of protein concentration in BALF, the total cell number of BALF in the P5 group increased 4.66-fold compared to the untreated group. However, there was no significant difference in the number of cells and protein concentration in BALF between the LL-37 and P1 groups compared to the PBS group. The results indicate that administration with 40 μL GF-17 (2.4 mmol/L) causes pulmonary inflammation response.

### 3.5. Histology of Lung Sections

**Figure 5:**
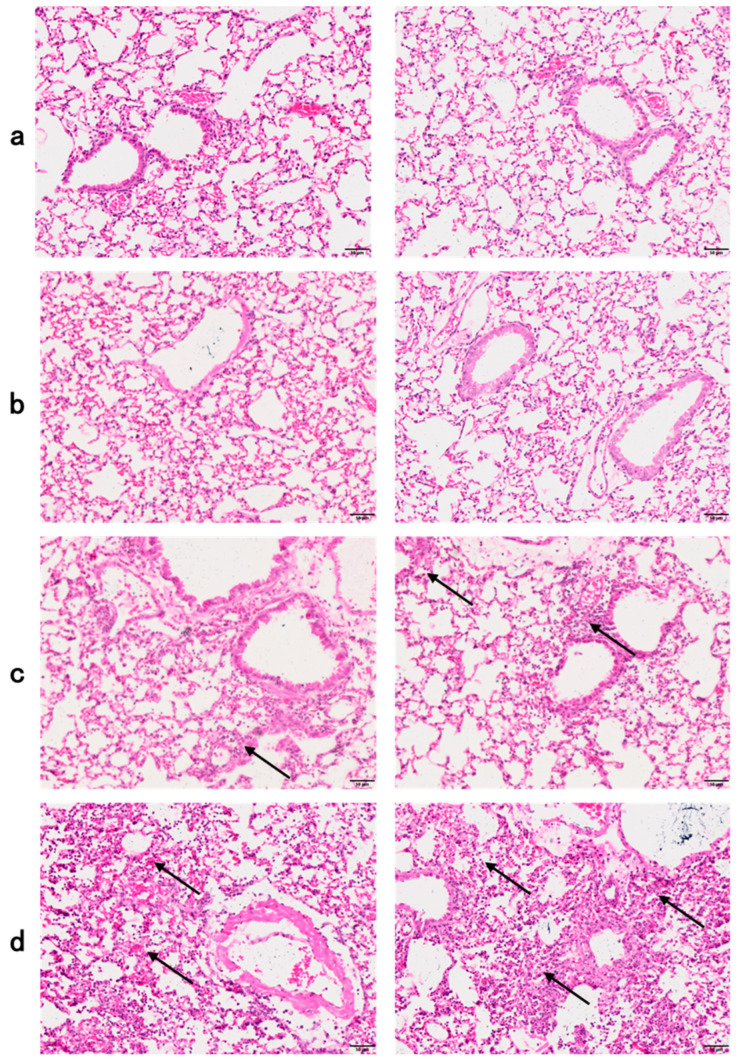
Histology of mouse lung sections. Neutrophils are marked with arrows. (**a**) PBS treated group. (**b**) LL-37 group. (**c**) P1 group. (**d**) P5 group. Scar bars, 50 µm.

Hematoxylin-eosin (HE)-staining sections of the paraffin-embedded tissues were prepared and used for histological analysis. Compared with PBS-treated mice (Figure 5a), there were no significant morphological changes in the lung tissue of the LL-37 group of mice (Figure 5b); however, group P1 had mild pulmonary interstitial edema, less alveolar exudation and neutrophil infiltration (Figure 5c), while group P5 had more severe pulmonary interstitial edema, alveolar exudation and neutrophil infiltration, and more severe lung tissue damage (Figure 5d). This also indicates that as the concentration of GF-17 increases, lung infection worsens, which is the pathological basis for the increase in alveolar lavage fluid protein concentration.

## 4. Discussion

AMPs represent a promising antibiotic alternative to overcome infections caused by drug-resistant bacteria due to their distinctive antimicrobial mechanism compared with traditional antibiotics. To date, 96 AMPs are currently registered in clinical trials (preclinical or clinical stage) [18]. Most of them are employed for topical use on the skin, and only a small amount can be used for intravenous injection, which greatly limits their application scope. The development of antimicrobial peptides that can be used intravenously or through pulmonary aerosol inhalation can effectively expand the scope of the use of antimicrobial peptides. In this article, we established a standard experimental method for the toxicity evaluation of AMPs in vivo, which can help antimicrobial peptides be applied in the clinic as soon as possible.

Although the contributions of physicochemical parameters, such as net charge, helicity, hydrophobicity and amphipathicity, to the antimicrobial activity and cell selectivity of AMPs are equivocal, they are believed to be important for the antimicrobial activity of AMPs [27]. Perfect amphipathicity has often resulted in a simultaneous increase in bactericidal activity and cytotoxicity, and the disruption of the α-helical amphipathic structure appears to be related to strong antimicrobial activity, reduced haemolytic activity, and hence, an improved AMP therapeutic index [11,28,29,30,31]. In this study, the μH of GF-17 and LL-37 was 0.771 and 0.521, which meant that GF-17 possessed higher amphipathicity than LL-37. Indeed, GF-17 exhibited higher haemolytic activity and higher antimicrobial activity than LL-37. The hydrophobicity property of peptides was characterised by the retention time of data and determined by reversed-phase high-performance liquid chromatography (RP-HPLC). The hydrophobic-binding domain in a peptide’s secondary structure can affect peptide interactions with reversed phase matrices, particularly for amphipathic α-helical peptides [32]. Indeed, GF-17 exhibited increased retention times, which were 20.727 min compared with 11.125 min for LL-37 (Table 1). This finding may be attributed to GF-17′s high α-helicity and, thus, higher binding properties to the reversed-phase matrices.

The antibacterial activity of GF-17, the truncated peptide of LL-37, is clearly better than that of LL-37, which is several times higher than that of LL-37. However, the hemolytic activity of GF-17 is much higher than that of LL-37. The TI, calculated by the ratio of MHC (haemolytic activity) and MIC (antimicrobial activity), is a widely employed parameter to indicate the specificity of AMPs. Larger TI values indicate greater antimicrobial specificity. GF-17 has better antibacterial activity than LL-37, especially against the Gram-negative bacteria *Klebsiella pneumoniae*. However, GF-17′s TI is lower than that of LL-37, which is due to its high hemolytic activity.

Considering that the bactericidal mechanism of antimicrobial peptides is to disrupt bacterial cell membranes, it is not difficult to predict that they may also have the ability to destroy eukaryotic cell membranes, resulting in cytotoxicity. Eukaryotic cytotoxicity is one of the factors that hinders the clinical application of AMPs. Obtaining the maximum possible antimicrobial activity with minimum toxicity towards the host is an attractive direction for antimicrobial peptide research and development. 

Toxicity tests at the cellular level are a routine method used to reflect the in vitro eukaryotic cytotoxicity of antimicrobial peptides. Among them, the hemolysis test is more sensitive because there is no serum during the experiment, which has a great influence on the activity of antimicrobial peptides. Enzymes, serum, pH and specific ionic strength in vivo influence the activity of AMPs. However, the in vitro toxicity test cannot fully represent its toxicity in vivo. As shown in this study, there was no significant difference in the cytotoxicity of GF-17 on human normal lung epithelial cell lines compared to LL-37, indicating that even in eukaryotic cells, the cytotoxicity caused by GF-17 may be significantly different. Greco et al. studied the correlation between the hemolytic activity and cytotoxicity of the antimicrobial peptide and found that a generally higher tolerance was exhibited by the human cells in comparison to human erythrocytes. One of the main challenges and limitations of the haemolysis experiment is the numerous ways it can be performed. Small changes, such as the choice of buffer or amount of DMSO potentially needed to generate a stock solution of peptides, generate different values [33]. Establishing a method for the rapid and comprehensive evaluation of antimicrobial activity and the toxicity of AMPs in vivo is of great help for the development of candidate AMPs for clinical application. 

Antimicrobial peptides are mostly applied locally, and intraperitoneal injection often leads to the local accumulation of drugs at the point of injection. The concentration is too high, and after directly acting on the skin, it is impossible to obtain suitable samples for other tests except for pathological testing; the processing of pathological samples is cumbersome; and the interpretation of results is highly subjective.

There is no good model available to evaluate the in vivo antibacterial activity and cytotoxicity of antimicrobial peptides. ALI is associated with excessive mortality and lacks appropriate therapy. Qin established a sepsis-induced acute lung injury model and found that the peptide LL-37 and its analogy sLL-37 attenuated the progression of sepsis-induced acute lung injury by inhibiting neutrophil infiltration and migration through the FAK, ERK, and P38 pathways [34]. In other studies, lipopolysaccharide (LPS)- and oleic acid (OA)-induced ALI animal models were established to evaluate the effect of peptides on ameliorating ALI [35,36,37]. In this study, we used peptide-induced mouse ALI to evaluate the in vivo cytotoxicity of the peptide. The mouse ALI model was established by the intranasal inoculation of peptides. No death was observed in all the treatment groups. As shown in Figure 3, the blood routine examination results show that WBC and neutrophil counts decreased with the increasing concentration of GF-17, while no significant change was found in the WBC count of the LL-37 group. RBC and blood platelet count remained stable between the PBS and P1 groups but increased for group P5, which may be related to the dehydration and weight loss caused by the poor condition of the mice. Mice in group P5 suffered a body weight loss of about 2.3 g 24 h after treatment, indicating that GF-17 has strong toxicity. The protein concentration and the inflammatory cell number of BALF in the P5 group increased by 3.32-fold and 4.66-fold, respectively, compared to the untreated group. Typical features of ALI are the alveolar accumulation of protein-rich fluid and activated immune cells due to pulmonary endothelial barrier disruption and increased vascular permeability [38,39]. The results indicate that GF-17 can induce inflammatory reactions in the lungs, leading to inflammatory injury in the lungs. Similarly, the histological examination results of mouse lungs showed the infiltration of neutrophils in the lungs of GF-17-treated mice, and the number of neutrophils in the lungs of treated mice increased with the increase in the GF-17 dose. Neutrophils play a key role in the development of different forms of ALI, and neutrophil counts are positively correlated with disease severity. Activated neutrophils rapidly migrate to inflamed lung tissue, releasing toxic granular contents and generating neutrophil extracellular traps [40,41]. The results indicate that high doses of GF-17 could induce acute lung injury, resulting in the accumulation of inflammatory cells in bronchoalveolar lavage fluid and neutrophil infiltration in the lung tissue.

## 5. Conclusions

GF-17 is a truncated peptide of LL-37 with higher haemolytic activity. However, there was no significant difference in the cytotoxicity against human lung epithelial cells between the GF-17 and LL-37 groups. Establishing a method for the rapid and comprehensive evaluation of antimicrobial activity and toxicity of AMPs in vivo is of great help for the development of candidate AMPs for clinical application. In this study, peptides were administered to mouse lungs via intranasal inoculation. Both LL-37 and GF-17 did not affect the red blood cells and platelet count, but GF-17 at the same concentration decreased the white blood cells and neutrophil counts in blood routine examinations. GF-17 can also induce acute lung injury, resulting in the accumulation of inflammatory cells in bronchoalveolar lavage fluid and neutrophil infiltration in the lung tissue. It is worth noting that the average body weight of mice administered with 40 μL GF-17 (2.4 mmol/L) decreased by 2.4 g within 24 h, indicating that GF-17 has significant toxicity in mice. Therefore, we established an animal model for the rapid evaluation of the in vivo toxicity of antimicrobial peptides. This study only provides a preliminary method for the evaluation of the toxicity caused by antimicrobial peptides in the lungs. For antimicrobial peptides with promising clinical applications, a systematic evaluation of their acute and long-term toxicity is required.

## Figures and Tables

**Table 1:** Sequence and biophysical properties of LL-37 and its analogue GF-17.

	LL-37	GF-17
Sequence	LLGDFFRKSKEKIGKEFKRIVQRIKDFLRNLVPRTES.NH_2_	GFKRIVQRIKDFLRNLV.NH_2_
Net charge	4	6
FreqPolar	0.622	0.529
FreqNoPola	0.378	0.471
Val_angle M	4.044	4.845
Calculated mass MW	4493.32	2101.54
Observed mass MW ^a^	4493.3	2101.5
Hydrophobicity (H)	0.201	0.378
Hyd.Moment (µH) ^b^	0.521	0.771
tR (min) ^c^	11.125	20.727

^a^ Molecular weight (MW) as measured by mass spectroscopy (MS). ^b^ μH, mean relative hydrophobic moment calculated by HeliQuest. ^c^ tR, retention time measured by reverse-phase HPLC.

**Table 2:** Antimicrobial activities of LL-37 and its analogue GF-17 against a panel of bacteria.

Peptide	MIC (μmol/L) ^a^				
	*E. coli*	*P. aeruginosa*	*K. pneumoniae*	*S. aureus*	*S. epidermidis*
LL-37	1.56	12.5	100	100	100
GF-17	6.25	6.25	6.25	6.25	3.125

^a^ Minimum inhibitory concentrations (MICs) were determined as the lowest concentration of the peptide that prevented visible turbidity.

## Data Availability

The data presented in this study are available on request from the corresponding authors.
